# Effect of Customized and Prefabricated Healing Abutments on Peri-Implant Soft Tissue and Bone in Immediate Implant Sites: A Randomized Controlled Trial

**DOI:** 10.3390/jcm13030886

**Published:** 2024-02-02

**Authors:** Parima Chokaree, Pongsakorn Poovarodom, Pisaisit Chaijareenont, Pimduen Rungsiyakull

**Affiliations:** Department of Prosthodontics, Faculty of Dentistry, Chiang Mai University, Chiang Mai 50200, Thailand; parima_ch@cmu.ac.th (P.C.); pongsakorn_poova@cmu.ac.th (P.P.); pisaisit.c@cmu.ac.th (P.C.)

**Keywords:** customized healing abutment, dental implant, immediate implant

## Abstract

This study aimed to evaluate the effect of customized healing abutments compared to prefabricated healing abutments in immediate implant sites. Twelve patients requiring single immediate implant placement were divided into two groups: a prefabricated group received prefabricated titanium healing abutments, and a customized group received a polyetheretherketone (PEEK) customized healing abutments fabricated based on the individuals’ digital impressions. Outcomes, including peri-implant horizontal and vertical soft tissue alteration, bone level change, volume change, pain score, and pink esthetic score (PES) change, were evaluated at the 1-, 4-, and 6-month follow-ups compared to pre-extraction teeth. At the 1- and 4-month follow-ups, the customized group had a significantly lower buccal volume variation (BVv). At the 6-month follow-up, neither group showed any significant difference in the marginal bone change; however, the customized group had a significantly lower PES change and a lower pain score. In the anterior and premolar regions, the customized group showed the preservation of peri-implant buccal horizontal soft tissue and buccal volume, while in the molar regions, the preservation of papilla height and midfacial height was observed. The morphology of the customized healing abutment demonstrated a better trend in preservation of peri-implant soft tissue, esthetic outcomes, and lower patient discomfort in immediate implant sites.

## 1. Introduction

Healing abutments are components used to promote peri-implant tissue healing. Prefabricated healing abutments, which usually come in round and cylinder shapes, have been widely used due to their convenience and time-saving nature. However, their morphology often leads to an unnatural soft tissue profile, resulting in unfavorable esthetic outcomes that may require additional surgery and recontouring procedures [1]. Customized healing abutments aim to develop a custom emergence profile of peri-implant supporting tissue immediately after implant placement. The goal is to create a natural appearance and promote peri-implant soft and hard tissue maturation [2,3,4,5]. In immediate implant placement, customized healing has been developed with the idea of capturing existing patients’ soft tissue to create an esthetically pleasing and natural appearance after extraction, avoiding the gradual adjustment undertaken in provisional restoration. To achieve this, the customized healing abutment is modified in its dimensions and transmucosal area to guide peri-implant tissue into the proper shape. Moreover, it can also be used to protect or seal underlying bone-grafting materials in the immediate placement socket without the need for sutures and to avoid disconnection of the abutment, which may interfere with the osseointegration process [6,7]. This results in larger dimensions compared to prefabricated healing abutments, which typically come in a circular shape [1]. In the past, the method used to create customized healing abutments was conventional, involving the chairside addition of flowable composite on the temporary abutment to capture the socket profile [8,9,10,11]. However, this technique still required continuous disconnections of the prosthetic components, leading to possible damage to peri-implant soft tissues [12,13]. With advances in digital dentistry, it is now possible to fabricate customized healing abutments prior to implant surgery, which can be inserted on the same day after implant placement with minor adjustments [14,15]. In this process, prototypes for creating customized healing abutments, such as patient socket dimensions, a cross-sectional area at the cementoenamel junction (CEJ) level, or the outline of the final prosthesis contour, are needed [14,16,17]. Commonly used materials include polymethylmethacrylate (PMMA) and polyetheretherketone (PEEK) [8] due to their physical and mechanical properties as provisional restorations [18]. Studies have reported techniques with favorable soft-tissue outcomes, preserving soft tissue and bone around the implant when using PMMA and PEEK customized healing abutments in immediate and delayed implant sites [14,17,19,20]. Limited clinical studies comparing the differences between these healing abutment types have been reported. Perez, A. et al. [21] reported better preservation in papilla height with the use of customized healing abutments compared to prefabricated healing abutments. Beretta, M. et al. [22] revealed less pain measured during prosthesis insertion when using customized healing abutments compared to prefabricated healing abutments. However, clinical studies reporting differences in peri-implant soft tissue and hard tissue alteration with these treatment modalities compared to the use of prefabricated healing abutments are lacking. Therefore, the purpose of this study was to evaluate the effect of the morphology of customized healing abutments compared to prefabricated healing abutments on soft tissue, hard tissue, pain assessment, and esthetic evaluation in immediate implant sites. Additionally, we evaluated the effect of customized healing abutments in different tooth positions on peri-implant tissue compared to pre-extraction teeth.

## 2. Materials and Methods

### 2.1. Study Design

**Figure 1:**
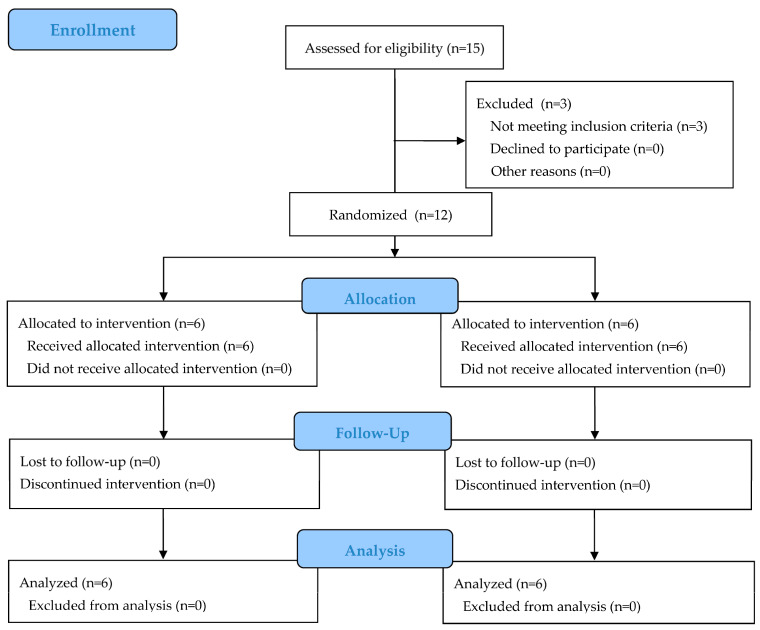
Clinical trial outline.

The present study comprised two parts. The first part was structured as a prospective controlled clinical trial with a parallel-group design to investigate outcome parameters associated with different types of healing abutments used as treatment methods for socket closure in flapless immediate implants in the maxillary or mandibular anterior and premolar regions. The second part was designed as a prospective clinical study to evaluate the same parameters using customized healing abutments in anterior and premolar regions compared to posterior immediate implant sites. The present clinical trial was conducted in compliance with the Declaration of Helsinki [23]. The study protocol was approved by the Faculty of Dentistry Human Experimentation Committee, Chiang Mai University, Chiang Mai, Thailand (No. 43/2022), and was registered at ClinicalTrails.gov (NCT06125418). In the first part, participants were divided into one control group (PG), which received a prefabricated titanium healing abutment, and one experimental group (CG), which received a customized PEEK healing abutment. The groups were assigned using balanced randomization (ratio 1:1). For the second part, all participants received customized PEEK healing abutments. A CONSORT 2010 checklist was utilized to ensure appropriate guidelines for the randomized trial study. The clinical trial outline is depicted in Figure 1. All clinicians involved in the study underwent one-day calibration training.

### 2.2. Participants

Patients were recruited from the Faculty of Dentistry at Chiang Mai University, and all participants provided informed consent before being enrolled in the trial. The treatment period spanned from June 2022 to June 2023. All patients requiring single-tooth extraction and immediate implant placement in the maxillary or mandibular anterior and posterior regions were recruited. Study participant selection was adapted from D. Fernandes et al. [24]. Patient inclusion criteria were as follows:

1. Patients ≥18 years of age.
2. Patients with a failing tooth requiring single implant placement therapy in the maxillary or mandibular arch in the anterior and posterior regions except the third molar area.
3. The failing tooth had adjacent and opposing natural teeth.
4. Sufficient mesial, distal, and interocclusal space for the placement of the implant and definitive restoration.
5. Had an intact socket wall before the extraction.
6. Had a type 1 extraction socket according to Juodzbalys et al. [25].
7. Had sufficient apical bone to place an immediate implant with a minimum primary stability of 30 Ncm.

Exclusion criteria were as follows:

1. Individuals were diagnosed with periodontal disease.
2. Medical and general contraindications for the surgical procedure.
3. Heavy smokers (>10 cigarettes/day).
4. An active infection at the implant site.

### 2.3. Interventions

**Figure 2:**
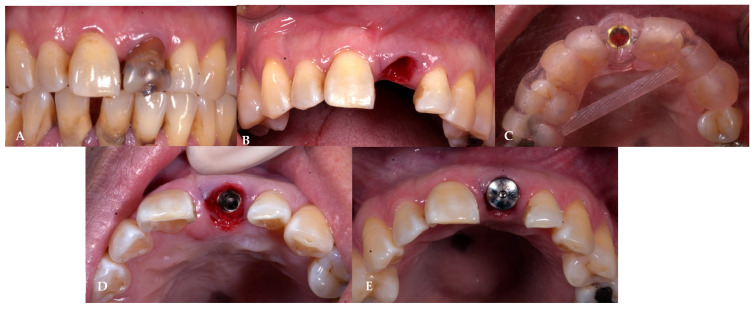
Surgical protocol in the PG group. (**A**) Pre-extraction tooth. (**B**) Atraumatic extraction. (**C**) Surgical guide try-in. (**D**) Implant placement with a surgical guide. (**E**) Prefabricated healing abutment insertion.

**Figure 3:**
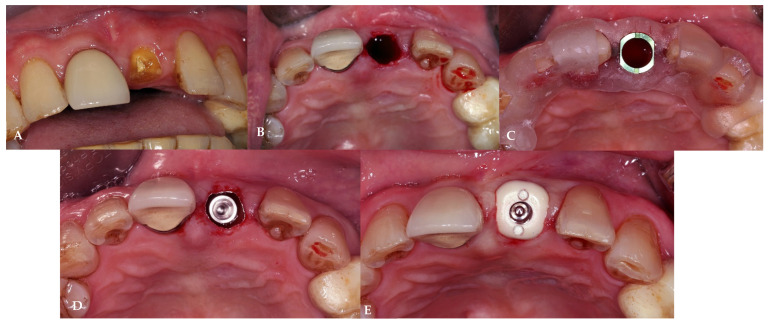
Surgical protocol in the CG group. (**A**) Pre-extraction tooth. (**B**) Atraumatic extraction. (**C**) Surgical guide try-in. (**D**) Implant placement with a surgical guide. (**E**) Customized healing abutment insertion.

**Figure 4:**
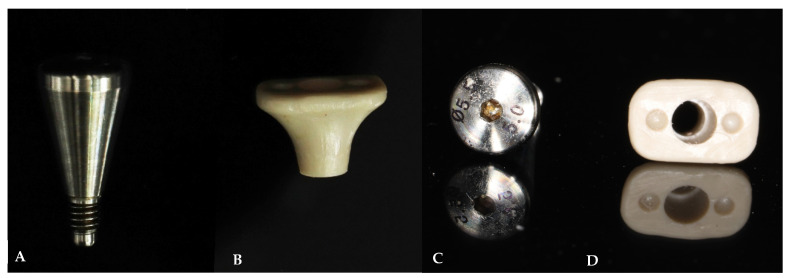
Healing abutments. (**A**,**C**) Prefabricated healing abutment. (**B**,**D**) Customized PEEK healing abutment.

The study protocol was adapted from D. Fernandes et al. [24] and A. Perez et al. [21] and was demonstrated in Figure 2 and Figure 3. Before the surgical procedure, all patients received at least one session of oral hygiene to provide a more favorable oral environment for wound healing. A clinician evaluated the eligibility of the tooth sites for immediate implant placement with delayed loading protocol, considering favorable clinical conditions and the absence of acute infection. After examination, patients underwent digital impressions using an intraoral scanner (CEREC Primescan, Sirona Dental Systems GmbH, Bensheim, Germany), and cone-beam computer tomography (CBCT) was performed for implant treatment planning. All enrolled patients received 2 g of amoxicillin (or 600 mg of clindamycin for those allergic to penicillins and cephalosporins) as pre-medication one hour before tooth extraction. Patients rinsed for 1 min with 0.2% chlorhexidine mouthwash (and twice a day for the following 3 weeks) and were treated under local anesthesia using 4% articaine with adrenaline at a ratio of 1:100,000 (Septanest, Septodont, UK). All surgical procedures were performed by well-trained surgeons. All tooth extractions were carried out with a flapless approach and with fine periotomes with the aim of reducing as much as possible the surgical trauma to the residual bone walls. Once the tooth was removed, the socket was carefully debrided and rinsed with saline solution. The clinician verified the integrity of the buccal wall and mesial and distal bony peaks with a periodontal probe to confirm the suitability of the treatment. The implant osteotomy site was then prepared according to the standard procedure with standard drills, maximizing the use of the apical bone. The bone-level implant (CMI IS-III Active implant, Neobiotech Co., Seoul, Republic of Korea) was placed with the platform located at the marginal level of the buccal bone wall or within 1 mm subcrestally, implying that the implant platforms were sub-crestal at mesial and distal sites. Vertical soft tissue thickness was confirmed with a periodontal probe to be at least 3 mm on palatal/lingual sites. The implant was placed in the correct 3-dimensional position with a surgical guide, engaging the palatal and apical bone to achieve high primary stability. After implant insertion, a gap of at least 2 mm between the inner cortical buccal bone plate and the implant surface was filled with bone grafting material. In situations where an immediate implant could not be placed, the patient was excluded from the study with spontaneous healing and received a delayed implant placement 3 months later. Once the implant was inserted and ensured to have primary stability, each patient received a healing abutment corresponding to the assigned treatment group as shown in Figure 4:

1. The prefabricated group (PG) received a prefabricated titanium healing abutment corresponding with the implant system and stabilized with single interrupted 6/0 polyamide sutures (SeralonTM, Serag-Wiessner, Nalia, Germany).
2. The customized group (CG) received a PEEK customized healing abutment allowing socket closure without sutures. PEEK was chosen for its biocompatibility and ease in the CAD/CAM fabrication technique [26].

All customized healing abutments were fabricated with dimensions based on the patient’s cross-sectional tooth at the cementoenamel junction (CEJ) level obtained from digital impressions. The emergence profile of the customized healing was designed to be gradually concave with an emergence angle not exceeding 30 degrees [27]. Each abutment was approximately 4 mm tall. All customized healing abutments were manufactured using CAD/CAM software (CEREC in LAB 5.0 MC XL, Sirona Dental Systems GmbH, Bensheim, Germany) and milled using a specific milling machine (Sirona MCX5, Sirona Dental Systems GmbH, Bensheim, Germany) before the implant placement appointment and were inserted after implant placement with a bone graft procedure. In anterior regions, the patients received provisional resin bonded crowns to the adjacent teeth on the same day as the implant surgery. The provisional restorations were set out of centric and eccentric occlusion and were removed after 16 weeks. Postoperative instructions included a soft diet, oral hygiene procedures, and rinsing with chlorhexidine 0.12% twice per day for 2 weeks. Systemic antibiotics (amoxicillin 1 g twice per day for 7 days) were prescribed as postoperative prophylaxis (for those allergic to penicillin, 300 mg clindamycin three times per day for 5 days). Analgesics (500 mg paracetamol three times per day) were prescribed as needed. Sutures were removed 10 days after surgery. Prosthetic procedures were performed 4 months after implant placement. A scanbody was connected to the implant, and a digital impression was acquired with the same intraoral scanner. A screw-retained provisional crown was delivered after 4 months of healing, and definitive restorations were inserted at the 6-month appointment, consisting of a screw-retained all-ceramic crown and a customized zirconia coping on a titanium base abutment. All patients entered a personally tailored supportive periodontal maintenance program, receiving oral hygiene instructions and scaling based on their individual needs. All surgical and prosthetic procedures were performed by experienced and well-trained clinicians who were not involved in the randomization process, follow-up evaluations, or statistical analysis. 

### 2.4. Clinical Observation and Data Acquisition

A digital impression was taken at four different time points in each group: T0 baseline (before tooth extraction), T1 (1st month after implant surgery), T4 (4th month with final impression process), and T6 (6th month before final prosthesis insertion). Each scan was performed by the operator within 30 s after the removal of the healing abutment to minimize soft tissue dimensional changes [28]. A periapical radiograph was taken at T0 and T6. Pain score and pink esthetic score (PES) measurements were performed at T6 (final restoration appointment). In all follow-up appointments, hygiene instructions were given to the patients, and periodontal care was executed when necessary. Clinical and radiographic controls were planned every year after implant insertion.

All digital models were exported from the intraoral optical scanner software in stereolithography (STL) format and were examined using specially designed software (Geomagic Control X, Geomagic, Inc., Cary, NC, USA). The T0 and T1, T0 and T4, and T0 and T6 STL files were superimposed, and a strict alignment was made into a common coordinate system. The final alignment was carried out through the best-fit alignment algorithm for a perfect match of digital models. 

### 2.5. Outcome Variables 

#### 2.5.1. Demographic Data

Patient information, including age, sex, and smoking habits, was recorded at baseline (T0).

#### 2.5.2. Soft Tissue Analysis

1. Horizontal tissue linear alterations (mm): mean buccal change (MBC) and mean total change (MTC).

**Figure 5:**
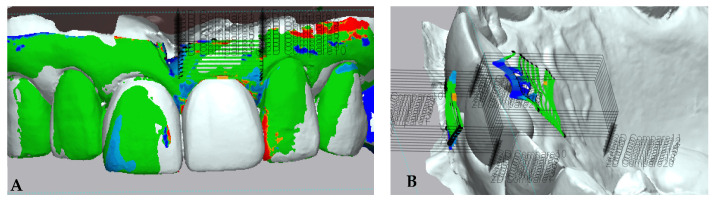
Assessment of linear horizontal tissue alteration. MBC measurement (**A**) and MTC measurement (**B**).

The digital analysis protocol was adapted from the method described by Fernandes, D. et al. [24] and is presented in Figure 5. After the superimposition study models, a color map was created to quantitatively analyze dimensional variations occurring in the surgical areas and surrounding tissues. Green represents areas where no 3-dimensional changes were found, while variations between yellow and red represent volume increase, and variations between light blue and dark blue represent a volume decrease.

A region of interest (ROI) composed of 10 section planes perpendicular to the coronal section of the tooth was computed at the buccal and palatal aspects of the ridge. These sections were set at the most apical point of the gingival margin and extended 5 mm apically. A line passing through the interproximal area limited mesial and distal boundaries of the ROI. The same ROI was used for each patient at different comparison follow-ups. The intersection of these sections with the superimposed models resulted in linear changes obtained in each area. The mean buccal change (MBC_T0–T1_, MBC_T0–T4_, MBC_T0–T6_) representing the buccal area and mean total change (MTC_T0–T1_, MTC_T0–T4_, and MTC_T0–T6_) representing the buccal and palatal aspects were calculated in millimeters (mm) using the multiple 2D compare function to evaluate the variations that occurred in the peri-implant area. A negative value refers to a reduction in horizontal measurement compared to baseline (T0).

2. Vertical linear tissue alterations (mm): mesial papillae height variation (mPHv), distal papillae height variation (dPHv), papillae height variation (PHv), and midfacial height variation (MFHv).

Papillae height and midfacial height at the baseline and at the 6-month follow-up were analyzed using computer software (Materialise Magics 23.01, Materialise, Leuven, Belgium). After precisely overlapping the T0 and T6 STL files in a common coordinate system, a standardized line (blue) was created connecting the two most apical points of the marginalgingiva of adjacent teeth, serving as a horizontal reference for the vertical measurements. Three measurements were computed in each STL file to calculate the mesial and distal papilla height and midfacial papilla height at T0 and T6. The mPHv, dPHv, MFHv, and PHv were obtained using the following formulas:

mPHv (mm) = mesial papilla height(mm)_T6_ − mesial papilla height(mm)_T0_ (1)

dPHv (mm) = distal papilla height(mm)_T6_ − distal papilla height(mm)_T0_ (2)

MFHv (mm) = midfacial papilla height(mm)_T6_ − midfacial papilla height(mm)_T0_ (3)

**Figure 6:**
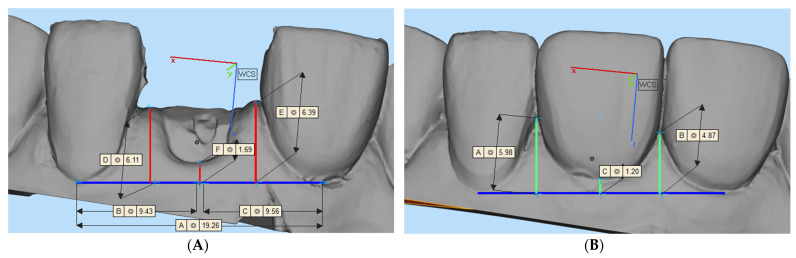
Assessment of midfacial mucosa and papillae height variation calculation. At baseline (**A**) and final prosthesis-insertion appointment (**B**).

The average between the mPHv and dPHv was the PHv. A negative value refers to a reduction in vertical height compared to baseline (T0). The vertical tissue measurements are presented in Figure 6.

#### 2.5.3. Hard Tissue Analysis

**Figure 7:**
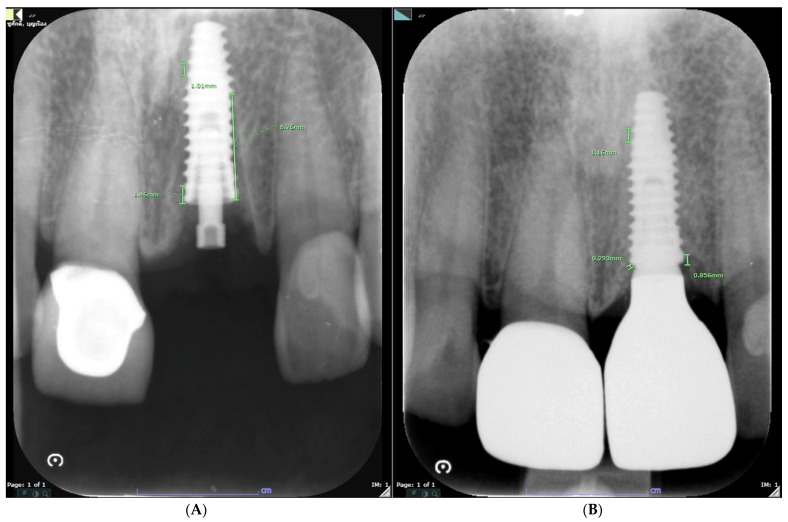
Assessment of hard tissue alteration. At baseline (**A**) and final prosthesis-insertion appointment (**B**).

Vertical bone loss via periapical radiograph was adapted from Perez, A. et al. [21] and is presented in Figure 7. The peri-implant marginal bone level was measured at baseline (T0) and then at the 6th month (T6) on intraoral radiographs at the mesial and distal aspects (m and d). It was defined as the distance between the reference point and the most apical point of contact between the implant surface and the bone, with the reference point being the fixture–abutment interface. The sign of the marginal bone level was considered negative when apical to the fixture–abutment interface. Digital intraoral periapical radiographs were taken (70 KVp, 7 mA) using a parallel cone technique with a digital sensor. A paralleling device and individualized bite blocks made of polyvinyl siloxane impression material (Impregum, Espe Dental AG, Seefel, Germany) were employed to standardize the X-ray geometry. Calibration was performed using the known thread pitch distance of the implants (0.8 mm). In cases where threads were not clearly visible on the radiographs, previously known values such as fixture diameter and length were used for calibration. Measurements were conducted using a Picture Archiving and Communication System (PACS). The marginal bone change (BC) was determined to the nearest millimeter using the following formula:

mBC= mesial marginal bone level_T6_ − measial marginal bone level_T0_ (4)

dBC= distal marginal bone level_T6_ − distal marginal bone level_T0_ (5)

A negative value of BC represents bone resorption; a positive value shows bone gain.

#### 2.5.4. Volumetric Analysis 

**Figure 8:**
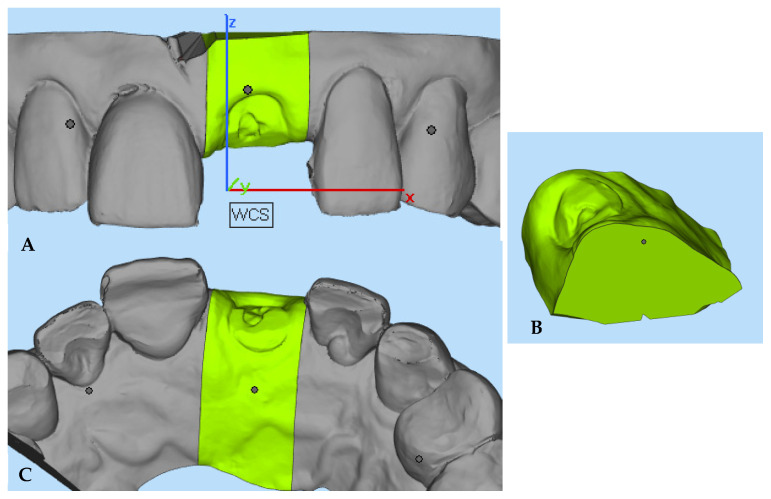
Assessment of volumetric calculation. ROI (**A**,**C**). TVt (**B**). Pink esthetic score and pain numerical rating scale.

Volumetric assessment is shown in Figure 8. The superimposed STL files were exported to another program (Materialise Magics 23.01, Materialise, Leuven, Belgium) for volumetric assessment. A 3-dimensional volumetric ROI was manually selected using the ‘cut’ or ‘punch’ function, with interproximal areas considered as mesial and distal limits. All cuts were consistently performed in the same areas in all digital models, ensuring uniform measurements across regions. The ‘Boolean’ function was employed to create STL files related to volume reduction and volume increase at different time points. Volumetric variation, considering both volume increase and volume reduction, was represented as buccal volume variation (BVv_T0–T1_, BVv_T0–T4_, and BVv_T0–T6_) and total volume variation (TVv_T0–T1_, TVv_T0–T4_, and TVv_T0–T6_) in relative percentages (%). The initial total volumes evaluated from each ROI at the buccal (BVt) and total (TVt) aspects were also computed for further comparison with volume variations at different appointments. These calculations enabled the creation of relative percentages of volume variations, which are essential for directly comparing different patients due to anatomical variances. All measurements were recorded to the nearest 0.01 mm.

Measurements were recorded at baseline (T0) and at T6 with the final prosthetic restoration. One examiner conducted all the clinical measurements, as reported below:

1. Pink esthetic score (PES from 0 to 14) comprises seven factors: mesial papilla, distal papilla, curvature of the facial mucosa, level of the facial mucosa, root convexity, soft tissue color, and texture at the facial aspect of the implant site [29]. PES was measured at baseline (T0) and at the final restoration insertion appointment (T6). The PES change was determined using the following formula:

PES change = PES_T6_ − PES_T0_ (6)

2. Pain numerical rating scale: After crown insertion, a questionnaire for the assessment of perceived pain was given to each patient. Each form contained the numerical rating scale (NRS), which consists of the personal evaluation of pain on a scale from 0 to 10 (0 is the minimum pain, and 10 is the maximum pain) [30]. The questionnaire was administered at three different study periods: at crown insertion (baseline) and after 2 and 24 h [22].

### 2.6. Statistical Analysis

The statistical analysis was performed using computer software (SPSS, Statistical Package for the Social Sciences, version 21.0, IBM Corp., Armonk, NY, USA) by an independent statistician who was not involved in the surgical procedure or study design. The established variables are presented as mean values and standard deviation. Participant characteristics such as age, sex, and smoking habits were evaluated using the chi-squared test, *t*-test, or Mann–Whitney test to examine possible significant differences between the initial characteristics of the groups.

Soft tissue outcomes, including linear, volumetric, midfacial, and papilla height change variables at different time points (T1, T4, and T6), mean marginal bone change, PES, and Pain NRS at different time points were evaluated with an independent *t*-test to identify differences for continuous non-paired variables. Within the customized group, changes in outcome variables at different time points (T1, T4, and T6) were evaluated with a paired *t*-test. All hypothesis tests were considered at the 5% level of significance.

### 2.7. Case Presentation

**Figure 9:**
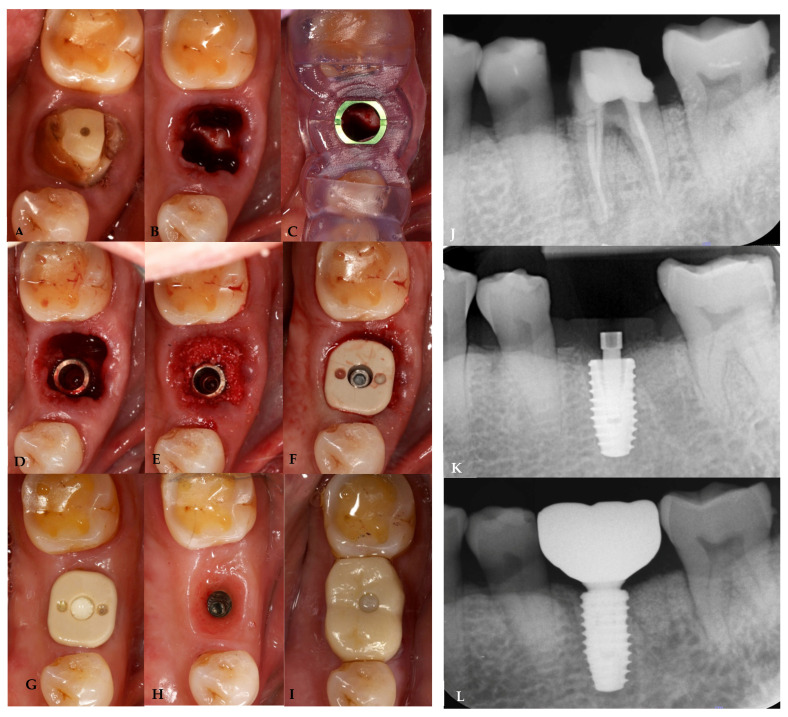
Clinical procedure. Fractured left lower first molar (**A**), after extraction (**B**), surgical guide try-in and protocol drill (**C**), immediate implant placement with simultaneous filling of the gap around implant with grafting material (**D**,**E**), customized healing abutment insertion (**F**), 4 months of healing (**G**,**H**), final prosthesis insertion 6 months after implant placement (**I**). Periapical radiograph of the pre-extraction tooth (**J**), healing abutment insertion (**K**), and final prosthesis insertion (**L**).

A 59-year-old systemically healthy, non-smoking man presented to the graduate prosthodontic clinic at the Faculty of Dentistry, Chiang Mai University, with a complicated crown-root fracture on the previously root-canal-treated mandibular left first molar. The patient expressed the wish to have the tooth replaced with a dental implant. Potential complications associated with the implant surgery were explained to the patient, and written consent was obtained. Oral examination, digital impression with an intraoral scanner, and CBCT were performed for implant planning. inLab CAD-CAM software (Sirona Dental Systems GmbH, Bensheim, Germany) was used to plan the implant position and to design and fabricate a customized healing abutment. On the day of surgery, the implant was placed following atraumatic extraction. A PEEK customized healing abutment was inserted without the need for suturing. The patient was recalled, and data were collected at follow-up periods, as mentioned above. A screw-retained final prosthesis was delivered 6 months after implant placement. All clinical procedures are presented in Figure 9.

## 3. Results

The patient distribution according to the CONSORT statement flow chart is reported in Figure 1. Initially, 25 patients were enrolled in the present trial. After tooth extractions, 3 out of 25 extraction socket sites did not have an intact buccal bone wall and were deemed unsuitable for immediate implant placement, resulting in their exclusion from the study. In total, 12 patients requiring anterior or premolar implant reconstruction were randomized into the two experimental groups and received their allocated treatment. Another 10 patients in need of a single implant placement in posterior teeth received a customized healing abutment. In each group, there were four patients who needed implant treatment in the maxillary anterior region and two patients who needed implant treatment in the mandibular premolar region. Patient demographic data are presented in Table 1.

There were 9 females and 13 males, ranging in age from 48 to 74, with a mean age of 58.5 years at the time of implant placement. Of these, 17 patients (75%) reported being non-smokers, and 5 patients (25%) reported being light smokers (consuming fewer than 10 cigarettes/day). At baseline, no significant differences were observed between the two groups. No implants were lost during the 6 months, resulting in an overall cumulative implant success rate of 100%.

### 3.1. Soft Tissue Parameters

#### 3.1.1. Horizontal Linear Tissue Alteration (mm): MBC and MTC

Linear peri-implant tissue variations from baseline to the 6-month follow-up are presented in Table 2. When comparing the types of healing abutments, both the PG and the CG groups demonstrated non-significant differences in all parameters. However, better trends were observed in the CG group. At T1, MBC was −0.27 ± 0.70 mm in the CG group and −0.44 ± 0.53 mm in the PG group, while mean total change (MTC) showed a linear alteration of −0.30 ± 0.54 mm in the CG group compared with −0.50 ± 0.28 mm in the PG group (*p* = 0.644 and *p* = 0.480, respectively).

At T6, the CG group exhibited less linear alteration than the PG group in both MBC and MTC, although no statistical significance was detected. MBC was −0.65 ± 0.62 mm in the CG group and −0.45 ± 0.58 mm in the PG group, while MTC was −0.53 ± 0.43 mm in the CG group compared to −0.61 ± 0.42 mm in the PG group (*p* = 0.544 and *p* = 0.739, respectively). When comparing at different time points, linear tissue alteration seemed to occur in both the PG and the CG groups. This was indicated by a significant change in MBC at T4 and T6 (*p* = 0.028 and 0.032 in the PG group, and *p* = 0.034 and 0.012 in the CG group, respectively). 

#### 3.1.2. Vertical Linear Tissue Alterations (mm); mPHv, dPHv, PHv, and MFHv

Papilla height variations at the 6-month follow-up were computed and are shown in Table 2. No statistical differences were found in any variables related to midfacial mucosa and papilla height variation. At T6, the CG group exhibited more apical migration of midfacial mucosa compared to the PG group (−0.61 ± 0.86 mm and −0.05 ± 0.64 mm, respectively). In contrast, both papillae sites showed less variation in the CG group compared with the PG group. Vertical tissue alterations of −0.85 ± 0.65 mm at the mesial site and −0.60 ± 0.75 mm at the distal site in the CG group contrasted with height variations at the mesial and distal papilla of −1.13 ± 0.51 mm and −1.23 ± 0.83 mm, respectively, in the PG group. At T6, the PG group demonstrated significant vertical tissue alterations in every variable (*p* = 0.008, 0.029, 0.007, and 0.875 for mPHv, dPHv, PHv, and MFHv, respectively), while the CG group showed significant changes in all variables except dPHv (0.023, 0.022, and 0.143 for mPHv, PHv, and MFHv, respectively).

### 3.2. Hard Tissue Parameters

#### Marginal Bone Change (mm); mBC and dBC

The peri-implant marginal bone change from implant placement to the 6-month follow-up is reported in Table 2. The mean marginal bone loss at the 6-month follow-up showed no statistical difference in either group. 

### 3.3. Volumetric Alterations

Volumetric alterations from baseline to the 6-month follow-up are shown in Table 2. The volumetric analysis revealed less change in BVv in the CG group; however, no statistical significance was detected.

### 3.4. Esthetic and Patient Satisfaction

#### 3.4.1. Pink Esthetic Score (PES)

The mean PES score change for the CG group was less than that for the PG group (−0.333 ± 1.51 and −2.75 ± 0.96, respectively), and the result showed statistical significance.

#### 3.4.2. Pain Numerical Rating Scaling (Pain NRS)

The pain numerical rating scale at the time of prosthesis insertion also revealed significant differences (*p* = 0.003) between the CG and PG groups (0.6 ± 0.89 and 3.4 ± 1.14, respectively). 

## 4. Discussion

The aim of the present study was to compare the effect of different healing abutments on peri-implant tissue in immediate implant sites. It has been reported that peri-implant tissue formation occurs immediately after implant placement, and the stability of both peri-implant soft and hard tissues is considered crucial for the long-term success of implant treatment [31,32,33]. Peri-implant soft and hard tissue stability and architecture after implant placement could be influenced by factors such as soft tissue quality and quantity, the type of surgical procedure, and the design of the prosthesis and abutment [34,35,36,37,38]. In this study, we focused on three major differences between prefabricated and customized healing abutments, including abutment dimensions, the abutment emergence profile, and the emergence angle.

1. Dimensions: The customized healing abutment has the patient’s socket size dimensions and is designed based on the cross-section of the patient’s tooth at the CEJ level from the digital intraoral impression and CBCT data. Prefabricated healing abutments, however, are circular in shape and vary in size from 5.0 to 5.5 mm in the anterior and premolar regions. Healing abutments of 6.0–6.5 mm were placed in the molar areas.
2. Macrogeometry: The customized healing abutment features a concave transmucosal design, whereas the prefabricated healing abutment has a straight transmucosal design.
3. Emergence angle: The emergence angle is defined as the angle between the average tangent of the transitional contour relative to the long axis of a tooth. Katafuchi et al. [27] recommended not exceeding a 30° emergence angle value to maintain soft tissue health in the transition zone. The dimensions of the customized healing abutments were larger than those of the prefabricated healing abutments. Therefore, the emergence angles of the customized healing abutments were wider, ranging from 20 to 30 degrees, while the prefabricated healing abutments have an angle of 11–15 degrees according to the manufacturers.

Considering dimensions, the customized healing abutments had a larger size, while the prefabricated healing abutments had a circular shape with a smaller size, leading to space around the socket, especially on the bucco-lingual aspect, necessitating the need for sutures. It has been noted that the smaller design of prefabricated healing abutments makes them incapable of adequately supporting peri-implant soft tissue architecture and mimicking a natural profile [1]. Our results align with this observation, as MTC at the 6th month was more pronounced in the prefabricated group, although the difference did not reach a significant level (−0.3557 ± 0.227 mm in the customized group and −0.607 ± 0.421 mm in the prefabricated group). This result was attributed to the design of the customized healing abutment, aiming to mimic the natural root contour at the CEJ level. This design is believed to preserve the pre-extraction soft tissue contour by allowing peri-implant tissue attachment and maturation. Our results differ from those of a previous study [24], where the authors observed more, though not significantly, MTC in the customized group at the 12-month follow-up. In that study, customized healing abutments were compared to the use of a collagen matrix with sutures instead of prefabricated healing abutments. The disparities in follow-up time points and the fact that the previous study compared the use of customized healing abutments with a collagen matrix make direct comparisons challenging.

In terms of macrogeometry, the concavity in the customized group was designed to gradually diverge from the fixture–abutment interface, finishing at the CEJ level. This results in more space at proximal sites, which may favor the stability of the papilla height compared to the straight profile. This is evident in PHv, where the customized group showed less reduction compared to the prefabricated group at the 6th month (−0.7258 ± 0.540 mm in the customized group and −1.179 ± 0.5238 mm in the prefabricated group). While the results did not reach statistical significance, they suggest a trend indicating that customized healing abutments might better preserve the papilla level, particularly in the case of the distal papilla, where tissue alterations were reduced by half in the customized group (−0.600 ± 0.750 mm and −1.232 ± 0.829 mm for the customized and prefabricated groups, respectively). This finding aligns with those of some authors [21] who observed that the use of customized healing abutments led to an improvement in the gingival papilla, while prefabricated healing abutments resulted in a reduction in papilla height through the papilla index (PI). These authors reported a significant improvement in both mesial and distal PI in the customized group compared to the prefabricated group at the 12-month follow-up. Another study [24] revealed less variation in papilla height in the customized group at the 6-month follow-up. However, in that study, the customized healing abutments were compared to the use of a collagen matrix with sutures instead of prefabricated healing abutments. Additionally, Dib-Zaitum, I. et al. [39] reported the effect of concave transmucosal abutments with better peri-implant soft tissue attachment. The authors concluded that abutment morphology could affect peri-implant tissue attachment more than surface treatment. Another study in dogs [40] also suggested that a concave transmucosal profile led to longer connective tissue attachment and less bone resorption compared to a straight profile. Moreover, a systematic review and meta-analysis [41] demonstrated the effect of concave transmucosal abutments in better preserving marginal bone levels than parallel/divergent implant transmucosal profiles. However, the present study revealed a non-significant difference in marginal bone change after 6 months. Therefore, according to our study, the concave transmucosal profile seems to influence papilla height preservation rather than marginal bone alteration.

Regarding the emergence angle, the wider emergence angle in the customized group was designed in conjunction with a larger dimension. This results in the possibility of mimicking the natural root contour, which may aid in the preservation of soft tissue architecture. As we observed, there was a significant reduction in BVv in the customized group in the 1st and 4th months. Our results align with a study [24] where BVv was less pronounced in the customized group compared to the collagen matrix group at the same follow-up periods. These findings could be beneficial in peri-implant tissue preservation, since some studies reported significant volumetric reduction following tooth extraction in the first 3 months [42,43]. However, it is important to note that volume alteration did not differentiate between hard and soft tissue volume. This variation might occur in both soft and hard tissue, as tissue alteration can be expected following tooth extraction [44]. While a wide emergence angle seems to preserve tissue volume, this angle may exert more pressure, pushing the midfacial level more apically. In contrast, in the prefabricated group, in which there is a smaller emergence angle, there is more room for the coronal migration of tissue on the buccal site. This is evident in MFHv, where the customized group showed reduction, while the prefabricated group exhibited slight coronal tissue migration at the 6-month follow-up (−0.608 ± 0.857 mm and 0.048 ± 0.641 mm, respectively). This trend is consistent with previous studies where the customized group revealed more reduction compared to the prefabricated healing abutment group [21,24]. In those studies, there was no information about emergence angles. Other studies [45,46] also reported esthetic complications involving midfacial mucosal recession of 0.27–0.54 mm after 1 year in function in cases of immediate implant placement. Since the coronal part of the tooth socket comprises bundle bone, which typically resorbs following tooth extraction, this leads to the subsequent apical migration of soft tissue. An FEA study [47] suggests that an implant abutment configuration with a taper of less than 10 or greater than 30 degrees could result in excessive pressure in the buccal gingival peri-implant region. Moreover, a study [40] in dogs revealed a wider straight emergence profile with a 45-degree angle, resulting in decreased peri-implant soft tissue height compared to a narrow emergence profile with a 15-degree angle. However, in this study, all healing abutments had a straight transmucosal design. From the present study, the customized healing abutment with a wider emergence angle than the prefabricated healing abutment revealed better trends in the preservation of peri-implant buccal soft tissue volume.

Our study revealed that in the fabrication of a customized healing abutment, all the factors mentioned above are correlated with each other. In immediate implant treatment, the purpose of a customized healing abutment is to mimic the preexisting tooth socket. Therefore, the dimensions must match the socket size. This leads to a larger emergence angle compared to a prefabricated healing abutment, where dimensions are usually slightly larger than the fixture diameter. The concavity of the emergence profile is gradually concave from the implant fixture–abutment interface to the CEJ level. Thus, we believe all these factors together influence peri-implant tissue responses. Prosthetic-driven planning ensured that all implants were placed in the correct three-dimensional positions with surgical guides. In this study, all implants used had a platform-switching design and were placed 0.5–1 mm subcrestally to engage apical and palatal/lingual bone with approximately 4 mm of vertical soft tissue thickness, which has been reported to maintain peri-implant marginal bone levels [48,49], allowing optimum support and stability of peri-implant soft and hard tissue [50]. Gaps between the implant and the buccal wall were filled with bone substitutions, as the study reported that simultaneous grafting following immediate implant placement revealed less horizontal and vertical alteration [51,52,53]. Moreover, all patients had intact socket walls after tooth extraction with a buccal plate thickness of at least 1 mm, a factor believed to influence the preservation of peri-implant tissue volume [24]. Both groups showed the ability to preserve mesial and distal marginal bone levels at the 6-month follow-up despite the types of customized healing abutment (mBC = 0.283 ± 0.465 mm and dBC = 0.359 ± 1.274 mm in the customized group and mBC = 0.428 ± 0.867 mm and dBC = 0.158 ± 0.353 mm in the prefabricated group). Therefore, customized healing abutments seem to influence peri-implant soft tissue rather than hard tissue variation. However, a longer follow-up period is still needed.

The positive trend in tissue preservation with the utilization of a customized healing abutment may result in an improvement in esthetic outcomes. In the present study, we employed PES change [29] for esthetic assessment, a widely used and objective method measured by a professional examiner based on defined criteria for evaluating soft tissue around a single implant. A minor negative PES change between the pre-extraction tooth and the final prosthesis was observed in the customized group, while the prefabricated group demonstrated a significant negative PES change (*p* = 0.002). A previous study reported a similar trend favored in the customized group [47,54]; however, the result did not reach a significant level. Therefore, the present study has highlighted varying tissue responses associated with the use of different healing abutments. While some parameters did not demonstrate statistically significant differences, there were noticeable trends favoring the customized group, which might impact esthetic appearances. The summary of significant differences between the utilization of prefabricated and customized healing abutments is reported in Table 3.

Regarding tooth position, our results show that in the customized group, MBC and MTC were not significant in the anterior and premolar regions (*p* = 0.108 and 0.095, respectively). We believe that the design of the customized healing abutment and the implant position in the anterior regions—where implant placement is recommended as being more palatal/lingual to engage bone—play important roles. Two factors result in more room for soft tissue on buccal sites, resulting in soft tissue stability. While posterior tooth implants are usually placed to engage septal bone, the implant is typically positioned close to the socket center, resulting in a smaller space on buccal sites. Therefore, the morphology of the customized healing abutment seems to preserve peri-implant soft tissue at 6 months on the buccal sites in the anterior and premolar regions. For vertical tissue alteration, a customized healing abutment could preserve the distal papilla in all regions. However, for mesial aspects, a significant difference was revealed in the anterior and premolar group, while the posterior results did not reach a significant level (*p* = 0.023 and 0.082, respectively). Further studies may be conducted to create an appropriate design for customized healing abutments for each tooth position that provide optimum peri-implant tissue stability.

It is important to note that, in addition to the morphology of healing abutments, several other factors could affect peri-implant surrounding tissue responses, including abutment type, implant placement depth and position, sites of implant placement, and vertical mucosal thickness. An FEA study demonstrated the direct effect of implant abutment types and implant placement depth on peri-implant bone remodeling [33,55]. Moreover, studies have reported the importance of implant positioning in peri-implant soft tissue stability [56,57]. In our study, a prosthetically driven approach was employed to ensure that implants were placed in the correct 3D position. All implants were placed using a surgical guide at 3 mm apical to the ideal position of the final prosthesis gingival margin. This allowed approximately 4 mm of vertical tissue thickness, which is reported to maintain the crestal bone level without developing peri-implant deep probing depth [48,58]. Furthermore, different implant locations refer to different hard and soft tissue qualities, which could affect outcome parameters [32,59]. In our study, both groups consisted of four anterior maxillary implants and two premolar mandibular implants. Thus, we believe that the same distribution of cases could minimize the effects of implant location on the outcomes. The primary limitation of this study is the restricted follow-up duration and the relatively small patient sample size. Collecting prospective data from a more extensive patient population over a longer observation period will strengthen the significance of our findings and provide a more comprehensive assessment of their applicability in a broader context. In this study, we have introduced objective protocols for evaluating peri-implant tissue, encompassing both hard and soft tissues, esthetics, and patient satisfaction. These protocols open the door for further investigations into the effects of customized healing abutments with varying parameters, such as the emergence angle of the abutment or the material used for customized healing abutments, as the study has indicated that these factors have the potential to influence the behavior of hard and soft tissue around the implant [60,61].

## 5. Conclusions

Customized healing abutments demonstrated different effects on peri-implant tissue compared to prefabricated healing abutments in immediate implant sites. The customized group demonstrated the preservation of buccal volume at the 1- and 4-month follow-ups compared to the prefabricated group. Neither group showed a difference in the preservation of marginal bone in the 6-month follow-up. The customized group exhibited a significantly lower PES change and a lower pain score than the prefabricated group.

Customized healing abutments showed different outcomes in different tooth positions. In the anterior and premolar regions, the customized group demonstrated the preservation of horizontal buccal tissue contours, while the posterior regions showed the preservation of mesial and distal papilla height at the 6-month follow-up.

## Figures and Tables

**Table 1:** Patients’ demographic data.

Demographic Data	Anterior and Premolar			Molar
	Customized Group	Prefabricated Group	*p*-Value	
Patient (n)	6	6		10
Implant location (maxilla/mandible)	(4/2)	(4/2)		(3/7)
Implant site (incisive/premolar)	4/2	4/2		
Age (years)	58 ± 6.85	60 ± 2.83	0.539	57 ± 3.46
Sex (%male/%female)	3/3 60%/40%	(4/2) 80%/20%	0.444	6/4

**Table 2:** Outcome variables.

Variables			Prefabricated Group					Customized Group				
			Anterior and Premolar		*p*-Value (Pre-Post)	Anterior and Premolar		*p*-Value (between Group)	*p*-Value (Pre-Post)	Molar		*p*-Value (Pre-Post)
			$\overset{-}{x}$	SD		$\overset{-}{x}$	SD			$\overset{-}{x}$	SD	
Soft tissue	Horizontal	MBC_T0–T1_	−0.442	0.537	0.139	−0.0754	0.509	0.332	0.786	−0.668	0.816	0.101
		MBC_T0–T4_	−0.490	0.587	0.135	−0.5388	0.340	0.867	0.012 *	−0.791	0.686	0.005 *
		MBC_T0–T6_	−0.453	0.578	0.154	−0.3507	0.439	0.745	0.108	−0.883	0.701	0.005 *
		MTC_T0–T1_	−0.491	0.280	0.017 *	−0.0889	0.405	0.121	0.690	−0.715	0.653	0.044 *
		MTC_T0–T4_	−0.613	0.406	0.028 *	−0.6817	0.576	0.828	0.034 *	−0.769	0.526	0.001 *
		MTC_T0–T6_	−0.607	0.421	0.032 *	−0.3557	0.227	0.237	0.012 *	−0.755	0.572	0.004 *
	Vertical	mPHv	−1.126	0.5129	0.008 *	−0.8517	0.647	0.463	0.023 *	−0.343	0.516	0.081
		dPHv	−1.232	0.8285	0.029 *	−0.6000	0.750	0.217	0.107	−0.032	0.869	0.914
		PHv	−1.179	0.5238	0.007 *	−0.7258	0.540	0.194	0.022 *	−0.188	0.547	0.333
		MFHv	0.048	0.6411	0.875	−0.6083	0.857	0.192	0.143	−0.561	0.960	0.118
Volume		BVv_T0–T1_	−28.465	3.8417	0.000 *	−10.8490	13.710	0.027 *	0.212	−34.109	11.510	0.010 *
		BVv_T0–T4_	−44.609	13.244	0.020 *	−19.787	16.175	0.023 *	0.030 *	−33.947	20.082	0.001 *
		BVv_T0–T6_	−26.631	20.788	0.046 *	−13.618	16.256	0.273	0.095	−29.644	13.875	0.000 *
		TVv_T0–T1_	−20.757	6.625	0.002 *	−12.005	7.179	0.099	0.044 *	−26.351	13.134	0.011 *
		TVv_T0–T4_	−41.206	18.468	0.008 *	−15.339	12.317	0.021 *	0.028 *	−33.339	17.142	0.000 *
		TVv_T0–T6_	−22.984	18.059	0.047 *	−11.217	7.498	0.177	0.015 *	−29.982	13.207	0.000 *
Hard tissue	Marginal bone change	mBC	0.428	0.867	0.332	0.283	0.465	0.751	0.244	0.118	0.240	0.135
		dBC	0.158	0.353	0.374	0.359	1.274	0.544	0.471	0.098	0.482	0.514
PES	change		−2.750	0.957	-	−0.333	1.506	0.022 *	-	0.833	1.169	-
Pain		0	3.400	1.140	-	0.600	0.894	0.003 *	-	0.200	0.422	-
		2 h	1.000	0.707	-	0.000	0.000	0.013 *	-	0.000	0.000	-
		24 h	0.000	0.000	-	0.000	0.000	0.500	-	0.000	0.000	-

* Statistically significant change at the 5% level.

**Table 3:** Summary of significant different outcome parameters.

Significant Difference Parameters	Prefabricated Group	Customized Group	*p*-Value	Similar Studies
BVv_T0–T1_	−28.47%	−10.85%	0.027	Collagen matrix group = −9.75% Customized group = −4.76% (*p* = 0.043) [24]
BVv_T0–T4_	−44.61%	−19.79%	0.023	Collagen matrix group = −8.98% Customized group = −6.39% (*p* = 0.336) [24]
TVv_T0–T4_	−41.21%	−15.34%	0.021	Collagen matrix group = −7.65% Customized group = −6.63% (*p* = 0.597) [24]
PES change	−2.75	−0.333	0.022	Prefabricated group = −0.6 Customized group = 0 (*p* value not indicated) [21]
Pain at 0 h	3.40	0.60	0.003	Prefabricated group = 5.5 Customized group = 0.5 (*p* = 0.0001) [22]
at 2 h	1.00	0	0.013	Prefabricated group = 2.7 Customized group = 0 (*p* = 0.0002) [22]

## Data Availability

Data supporting the study findings are available from the corresponding author upon request.
